# Continuous Three-Dimensional Printing of Architected Piezoelectric Sensors in Minutes

**DOI:** 10.34133/2022/9790307

**Published:** 2022-07-11

**Authors:** Siying Liu, Wenbo Wang, Weiheng Xu, Luyang Liu, Wenlong Zhang, Kenan Song, Xiangfan Chen

**Affiliations:** ^1^School of Manufacturing Systems and Networks, Arizona State University, Mesa, AZ 85212, USA; ^2^The Polytechnic School, Arizona State University, Mesa, AZ 85212, USA; ^3^School for Engineering of Matter, Transport & Energy, Arizona State University, Tempe, AZ 85287, USA

## Abstract

Additive manufacturing (AM), also known as three-dimensional (3D) printing, is thriving as an effective and robust method in fabricating architected piezoelectric structures, yet most of the commonly adopted printing techniques often face the inherent speed-accuracy trade-off, limiting their speed in manufacturing sophisticated parts containing micro-/nanoscale features. Herein, stabilized, photo-curable resins comprising chemically functionalized piezoelectric nanoparticles (PiezoNPs) were formulated, from which microscale architected 3D piezoelectric structures were printed continuously via micro continuous liquid interface production (*μ*CLIP) at speeds of up to ~60 *μ*m s^−1^, which are more than 10 times faster than the previously reported stereolithography-based works. The 3D-printed functionalized barium titanate (f-BTO) composites reveal a bulk piezoelectric charge constant *d*_33_ of 27.70 pC N^−1^ with the 30 wt% f-BTO. Moreover, rationally designed lattice structures that manifested enhanced, tailorable piezoelectric sensing performance as well as mechanical flexibility were tested and explored in diverse flexible and wearable self-powered sensing applications, e.g., motion recognition and respiratory monitoring.

## 1. Introduction

Piezoelectric materials that could convert mechanical energy to electricity or vice versa [1, 2] have enabled diverse applications such as energy harvesting [2–4] and self-powered sensing [5–8]. A wide variety of inorganic ceramics, e.g., barium titanate (BTO) and lead zirconate titanate (PZT), and organic polymers, e.g., poly(vinylidene fluoride) (PVDF) and its copolymers (e.g., P(VDF-TrFE)), have attracted unprecedented attentions [9]. Nonetheless, neither the piezoelectric ceramics nor the piezoelectric polymers can simultaneously meet the demands from applications in flexible, wearable, or implantable electronics in terms of piezoelectric performance, mechanical flexibility, or ease-of-processibility [10–17]. Alternatively, incorporating piezoelectric nanoparticles (PiezoNPs) into flexible host matrices has been proven to be a satisfactory strategy in developing composites that possess both decent piezoelectric responses and tailorable, compliant mechanical performance [18, 19]. However, conventional manufacturing of piezoelectric devices is usually achieved by solution deposition (e.g., spin coating and dip coating) as well as chemical or physical vapor deposition (e.g., thermal depositing and magnetron sputtering) onto planar substrates for two-dimensional (2D) structures, which imposes severe limits on further geometric modifications for three-dimensional (3D) applications [20, 21]. To realize 3D-architected piezoelectric devices possessing enhanced piezoelectric performance and expand the piezoelectric applications into 3D realm, various additive manufacturing (AM) techniques, such as fused deposition modeling (FDM) [22, 23], inkjet printing [24], and projection micro-stereolithography (P*μ*SL) [25–29], have been explored. For example, Cui et al. reported utilizing P*μ*SL to print PZT-based piezoelectric composite structures with designed anisotropies and directional responses [25]. However, P*μ*SL still faces the inherent speed-accuracy trade-off owing to its stepwise layer-by-layer nature that limits the overall scalability in manufacturing sophisticated 3D parts with fine features [30, 31]. Moreover, due to the repeated delamination and movement, the printing inconsistencies and stair-stepping effects are significant, which subsequently deteriorate the mechanical integrity of the final parts [32]. On the contrary, recently developed continuous liquid interface production (CLIP) has the advantage to print delicate 3D structures possessing superb surface finish and homogenous mechanical properties at high speeds by adopting an oxygen-permeable window to eliminate the delamination process [33, 34].

**Figure 1:**
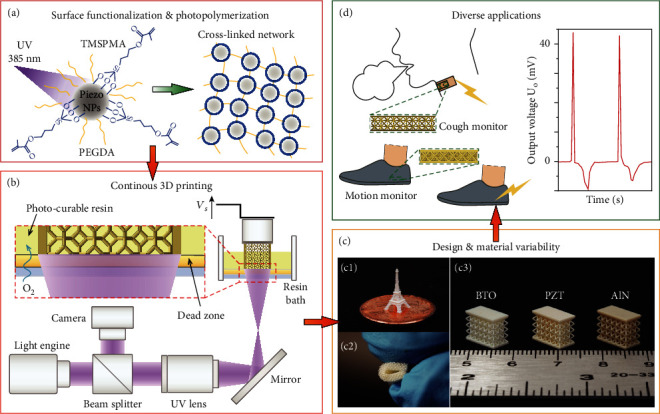
Schematic of rapid, continuous 3D printing of piezoelectric sensors. (a) Schematic illustration of the surface functionalization of PiezoNPs and subsequent photopolymerization. (b) Schematic illustration of the *μ*CLIP setup. (c) Optical images of piezoelectric composite structures, including (c1) a printed f-BTO Eiffel tower, (c2) a flexible f-BTO composite structure, and (c3) a set of PiezoNPs composite structures. (d) Proof-of-concept demonstrations for human body motion sensing and respiratory monitoring.

Herein, as schemed in Figure 1, we developed a serial of photo-curable resins with PiezoNPs, including BTO, PZT, and aluminum nitride (AlN), via the assistance of chemical functionalization process (Figure 1(a)). In the perspective of environmental toxicity and biocompatibility [35, 36], we selected BTO as a representative dopant because of its decent intrinsic piezoelectric performance as well as its lead-free, biocompatible nature [37, 38] and formulated resins with functionalized BTO (f-BTO) concentration up to 30 wt%. To print high-quality piezoelectric devices, a customized micro continuous liquid interface production (*μ*CLIP) method was developed (Figure 1(b)) [39]. Upon optimizations of the resins as well as the *μ*CLIP processing parameters, we successfully printed different piezoelectric structures at both micrometer and centimeter scale with unprecedented printing speeds of up to ~60 *μ*m s^−1^, and we confirmed that such high speeds can also be applied to PZT or AlN composites (Figure 1(c3)). We conducted systematic characterizations on the 3D-printed f-BTO composite structures in terms of their compositional, mechanical, and piezoelectric properties and verified their potentials under various flexible and wearable piezoelectric sensing scenarios (Figure 1(d)).

## 2. Results

### 2.1. Synthesis and Rheological Characterization of the f-BTO Resins

**Figure 2:**
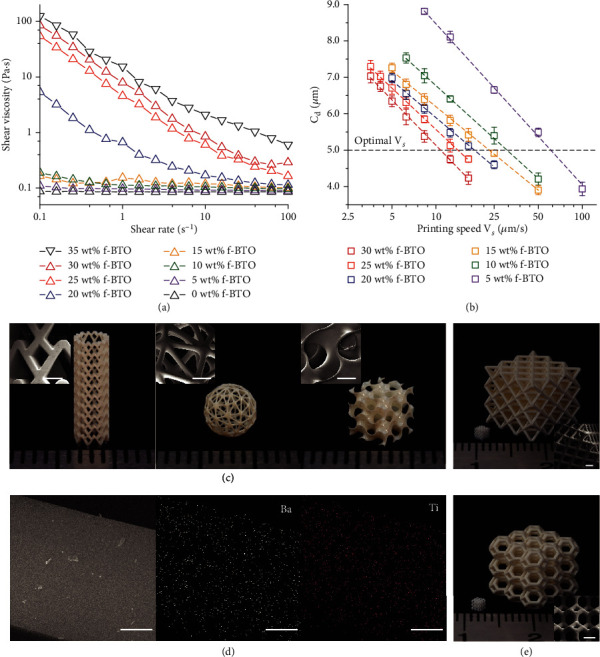
Optimized continuous printings of architected piezoelectric structures. (a) Measured shear viscosity of the resins with different f-BTO loadings. (b) Measured curing depth *C*_*d*_ versus logarithmic printing speed *V*_*s*_ of the f-BTO resins. (c) Optical images of the 3D-printed sophisticated structures. Inset: SEM images of the enlarged features (scale bar: 500 *μ*m). (d) SEM image and associated EDS elemental mappings of a round beam printed with the f-BTO resin (scale bar: 50 *μ*m). (e) Optical images of the 3D-printed micrometer- and centimeter-scale lattice structures. Inset: SEM images of enlarged features of the micrometer-scale lattice structures (scale bar: 250 *μ*m).

To avoid the agglomeration and precipitation of PiezoNPs from the resins and ensure homogeneous properties of the printed parts, we adopted 3-(trimethoxysilyl)propyl methacrylate (TMSPMA) to effectively functionalize the PiezoNPs, which enables steric hindrance that stabilizes the colloids. The stabilized resins can efficaciously suppress the formation of voids or phase separation between the polymeric networks and the PiezoNPs, both of which are detrimental to the piezoelectric performance. Furthermore, the functionalized PiezoNPs can be covalently bonded into the polymeric matrices via the vinyl groups of TMSPMA, forming an interfacial linkage between the compliant polymeric matrices and the stiff PiezoNPs, which effectively enhances the stress transfer for improved electromechanical performance (Figure 1(a) and in Materials and Methods) [25, 28, 40, 41]. Here, we selected commercially available BTO nanoparticles (with average particle size of 50 nm) with loadings up to 30 wt%. To achieve continuous printings of resins comprising increased f-BTO's concentration without sacrificing desired printing speeds and quality, nanoscale particles (also abbreviated as PiezoNPs before) are chosen over micron-scale powders to alleviate the phase separation between the piezoelectric fillers and the liquid polymeric matrix which would lead to disrupted printings, as well as to achieve finer features since large powders tend to block the incident UV light and are detrimental to desired printing resolution. More importantly, adopting PiezoNPs could boost the piezoelectric responses of the printed composites as there are more available binding sites on PiezoNPs due to much higher surface area to volume ratio [42]. Poly(ethylene glycol) diacrylate with average molar mass *M*_*n*_ of 700 (PEGDA 700) was selected as the host matrix because of its intrinsic compliance and biocompatibility [43–46]. To quantitatively characterize the photo-curable resins, rheological studies were conducted. Specifically, though the unfunctionalized 30 wt% BTO resin did not show visually noticeable agglomeration until ~2 d because of the fine crystallites (Figure S1), oscillation time sweep over 60 min (in Materials and Methods and Figure S2a) evidently indicates that surface functionalization fundamentally altered rheological characteristics of the 30 wt% resin from slurry-like, highly unstable mixture (quantified by tan(*δ*) < 1 and an 47.8% increasement) to stabilized colloidal suspension (quantified by tan(*δ*) > 1 and an insignificant change of 10.5%). This is because the lower tan(*δ*) value indicates the formation of large BTO nanoparticle clusters and their tendency to restrict the mobility of the viscose polymer molecular chains. The large value variation over time indicates unstable resin behavior as the large clusters are more prone to sediment, resulting in inconsistent resin composition. Furthermore, the viscosity of the unfunctionalized BTO resin is approximately 10 times higher than that of the f-BTO resin (Figures S2b), which is likely due to cluster jamming between the parallel plates, and this is essentially similar to the realistic scenario during the printings where unfunctionalized BTO nanoparticles rapidly agglomerate and sediment onto the transparent window, causing devastating scattering of the input UV light and inhibiting the continuous flow-in of resins, all of which will eventually lead to disrupted printings. Viscosity of the resin increased rapidly as f-BTO concentration went up (Figure 2(a)), and when the f-BTO concentration increased from 30 wt% to 35 wt%, shear viscosity showed a steep increase (nearly doubled from 7.93 Pa∙s to 15.42 Pa∙s at shear rate of 1 s^−1^) (Figure S2c). By combining the rheological studies with our preliminary tests, we selected 30 wt% f-BTO to be the upper limit of the resins, with which we can achieve satisfactory microstructures and piezoelectric performance without compromising the desired high-speed printings.

### 2.2. 3D Printing of Architected Piezoelectric Structures via *μ*CLIP

To yield rapid, high-resolution 3D printings of the functionalized resins (Figure 1(b) and in Materials and Methods), we have developed a speed working curve method to experimentally parameterize the optimal printing speeds of the customized *μ*CLIP system (Supplementary Section 1 and Figure S3) [47]. A substrate-assisted printing strategy was utilized to address the weak adhesion between the initial solidified layers and the building platform at high f-BTO concentrations (Supplementary Section 2 and Figure S4). The speed working curve model can be expressed as

(1) $$\begin{array}{c} C_{d}=D_{p}\times \ln\left(\frac{V_{c}}{V_{s}}\right), \end{array}$$

where *C*_*d*_ is the curing depth, *D*_*p*_ is the penetration depth of the input UV light, *V*_*c*_ is the threshold moving speed of the printing platform, and *V*_*s*_ is the actual moving speed of the printing platform. The measured *C*_*d*_ versus logarithmic *V*_*s*_ for resins with different solid loadings were plotted and fitted according to the underlying curing model in Figure 2(b) and Figure S5, which can guide the determination of the optimal printing speed *V*_*s*_. As the layer slicing thickness was 5 *μ*m, we can adopt a *V*_*s*_ of ~10 *μ*m s^−1^ even for the 30 wt% f-BTO resin, which allows the fabrication of a one-centimeter-long 3D structure comprising micron-scale features in ~15 min (Supplementary Movie 1), while it takes ~7 hr or even longer via a P*μ*SL system based on our previously reported work [31]. More importantly, the optimal *V*_*s*_ increases substantially as f-BTO wt% decreases due to the alleviated particle-induced light scattering as well as dropped viscosity. For example, the optimal *V*_*s*_ for 20 wt% f-BTO resin can be determined as 18.30 *μ*m s^−1^. Using the parameters determined above, sophisticated 3D structures, including a vascular stent, a hollow lattice ball, and a gyroid structure, were printed with the f-BTO resin (Figure 2(c)). The scanning electron microscopy (SEM) images reveal the smooth, stairstep-free surface morphology of the microscale features, and associated energy dispersive X-ray spectroscopy (EDS) mappings of the elements (i.e., Ba and Ti, with atomic ratio of 1.04 : 1) confirm the uniform dispersion of the f-BTO nanoparticles (Figure 2(d)). We also demonstrated that it is facile to yield 3D structures (e.g., octet-truss and Kelvin lattices) ranging from micrometer to centimeter scale (Figure 2(e)) or fabricate 3D structures comprising different PiezoNPs to meet diverse demands (Figure 1c3).

### 2.3. Characterizations of the 3D-Printed f-BTO Composites

**Figure 3:**
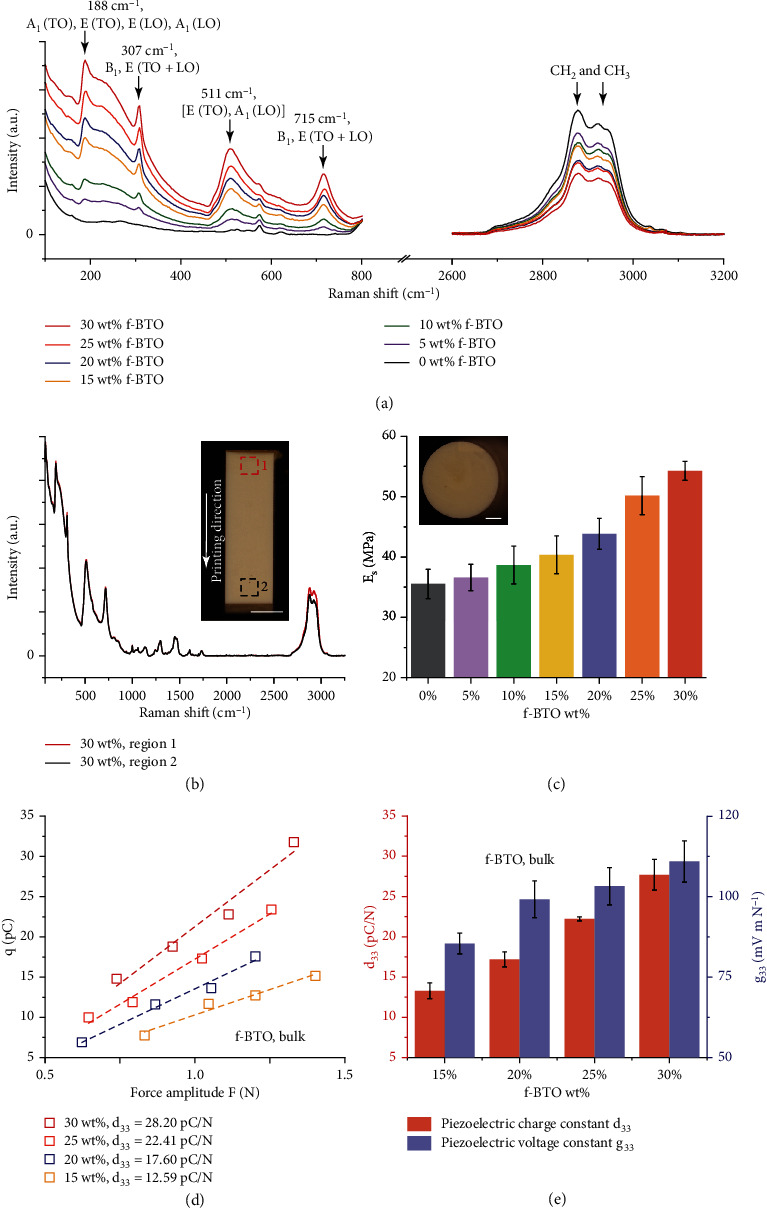
Characterizations of the 3D-printed piezoelectric composites. (a) Raman spectra corresponding to the characteristic peaks of f-BTO and the characteristic C-H_2_ and C-H_3_ peaks of PEGDA 700 [50] embedded within the 3D-printed composites. (b) Raman spectra obtained from the well-separated region 1 and region 2 of a 3D-printed 30 wt% f-BTO beam structure (scale bar: 1 mm). (c) Measured Young's modulus *E*_*s*_ of the 3D-printed f-BTO composites. Inset: optical image of the pillar structure used for compression tests (scale bar: 500 *μ*m). (d) One representative set of data of the measured charges *q* versus force amplitude *F* for the f-BTO bulk composites. (e) Extracted piezoelectric charge constant *d*_33_ and piezoelectric voltage constant *g*_33_ of the 3D-printed f-BTO composites.

**Figure 4:**
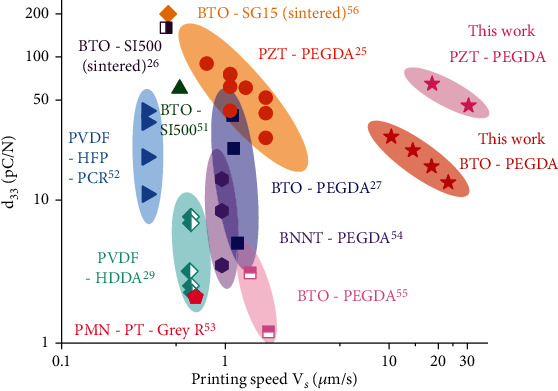
Comparison between this work and other reported works in terms of printing speed and *d*_33_ [25–27, 29, 51–56].

Raman spectroscopy (Figures 3(a) and 3(b) and in Materials and Methods) was first employed to analyze the compositions of the 3D-printed f-BTO composites. The results not only reveal the characteristic peaks corresponding to a mixture of cubic (188 cm^−1^, 511 cm^−1^ and 715 cm^−1^) and tetragonal (307 cm^−1^) BTO nanoparticles (Figure 3(a)) but also confirm the correspondence between peak intensities and the f-BTO (PEGDA 700) concentration [48, 49]. In detail, with the increase of f-BTO concentration (the concentration of PEGDA 700 decreases accordingly), the characteristic peaks of f-BTO increase and characteristic C-H_2_ and C-H_3_ peaks of PEGDA 700 decrease simultaneously. Moreover, the highly identical Raman spectra taken from two well-separated regions (3 mm spacing) along the printing direction helped confirm the compositional homogeneity throughout the entire structure as no leaching out of f-BTO nanoparticles happened during the printings because of the chemically stabilized resins (Figure 3(b)). Next, bulk Young's modulus *E*_*s*_ of the 3D-printed f-BTO composites was measured, which increases monotonically from 36.60 MPa to 54.27 MPa with respect to f-BTO concentration from 0 wt% to 30 wt%, thus verifying the compositional tunability upon the mechanical compliance of these f-BTO composites (in Materials and Methods and Figure 3(c)). Additional mechanical characterizations on the 3D-printed and thermally postcured f-BTO composites (in Materials and Methods and Figure S15) indicate that the piezoelectric composites were thoroughly polymerized during the printing process and that the elevated temperature during subsequent corona-poling treatments has little impacts on the mechanical properties of the samples. To quantify the piezoelectric performance, we established a noncontact corona-poling setup to polarize the 3D-printed structures and measured the piezoelectric charge constant *d*_33_ of the f-BTO composites via a calibrated piezoelectric charge characterization setup (in Materials and Methods and Supplementary Section 3, Figure S6, and Figure S8). One representative set of data on *d*_33_ of the bulk samples printed with 15-30 wt% f-BTO composites was plotted in Figure 3(d), which indicates the clear linearity between generated charges *q* and applied force amplitude *F*, as well as the monotonic increasement of *d*_33_ from 12.59 pC N^−1^ to 28.20 pC N^−1^ with f-BTO increasing from 15 wt% to 30 wt%. It is worth mentioning that the piezoelectric responses of the 3D-printed composites reach the upper boundary at a given loading concentration, which can be validated by additional measurements on the 30 wt% f-BTO composites that confirm the BTO nanoparticles were thoroughly functionalized for maximized output (Supplementary Section 4 and Figure S9). To further underline the impacts from the particle size on the piezoelectric performance, f-BTO composites were also printed with 100 nm f-BTO and characterized, and a significant decreasement of ~43.98% in *d*_33_ was observed from the side-by-side comparison (Figure S10), which is attributed to the predominant effect of increased surface to volume ratio as the average particle size reduces, thus enhancing the stress transfer by providing more available binding sites [42]. To assess the composites' suitability for sensing applications, we also explored the piezoelectric voltage constant *g*_33_, which is defined to quantify the electrical field induced per unit stress applied (in Materials and Methods and Supplementary Section 6 and Figure S11), and the bulk *d*_33_ (*g*_33_) values extracted from the printed f-BTO composites (Figure 3(e)) increase monotonically from 13.29 pC N^−1^ to 27.70 pC N^−1^ (*g*_33_ from 85.19 mV m N^−1^ to 110.71 mV m N^−1^) with respect to the f-BTO concentration. To demonstrate that the developed printing procedure is intrinsically versatile and can be generalized to other typical piezoelectric ceramics, we also succeeded in printing 10 wt% and 20 wt% f-PZT composites that come with remarkably enhanced piezoelectric performance (*d*_33_ = 64.87 pC N^−1^ and *g*_33_ = 269.47 mV m N^−1^ for the 20 wt% f-PZT composite; Figure S12), or f-AlN composites at similar speeds, both of which further manifest the capability of the developed procedure. In particular, the piezoelectric performance of the 3D-printed bulk f-BTO and f-PZT composites is comparable to pure piezoelectric materials such as PVDF or P(VDF-TrFE), but still not as good as the corresponding pure BTO and PZT ceramics (a summary of commonly utilized piezoelectric materials can be found in Table S1). The major advantage of the piezoelectric composites reported in this work is their ease-of-processibility by which they can be directly fabricated into architected piezoelectric structures. Overall, with the well-developed procedure, we are able to fabricate 3D structures with piezoelectric performance comparable to state-of-the-art P*μ*SL-based works at speeds that are at least one order of magnitude faster (Figure 4).

### 2.4. Characterizations of the 3D-Printed f-BTO Lattice Structures

**Figure 5:**
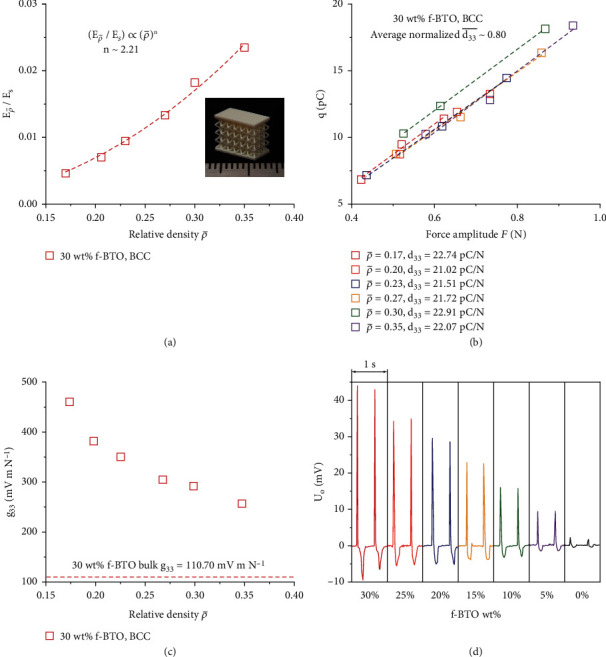
Characterizations of the 3D-printed f-BTO BCC lattices. (a) Relative modulus $\left(E_{\bar{\rho }}/E_{s}\right)$ versus $\bar{\rho }$ for the BCC structures printed from 30 wt% f-BTO resin. Inset: optical image of a 3D-printed BCC structure used in the characterizations. (b) Representative data of measured charges *q* versus force amplitude *F* of the 30 wt% f-BTO BCC structures with varying relative density $\bar{\rho }$. (c) Extracted piezoelectric voltage constant *g*_33_ of the 30 wt% f-BTO BCC structures versus $\bar{\rho }$. (d) Output voltage *U*_*O*_ measured from the BCC structures ($\bar{\rho }=0.17$) printed with f-BTO resins at a fixed force amplitude *F* = 0.85 *N*.

To show that one can obtain enhanced mechanical compliance and piezoelectric sensing performance for flexible sensing applications, we selected the body-centered cubic (BCC) lattice as a demonstration because of its superior mechanical compliance among commonly seen lattices [57]. We explored the mechanical and piezoelectric performance of 3D-printed BCC structures with varying relative density $\bar{\rho }$ (refer to Figure S14 and Table S2 regarding the geometrical parameters of the 3D-printed BCC lattices as well as Supplementary Section 5 and Figure S13a for the determination of $\bar{\rho }$). In detail, we extracted the modulus $E_{\bar{\rho }}$ based on the obtained stress-strain curves (Figure S13b), from which we calculated the relative modulus $\left(E_{\bar{\rho }}/E_{s}\right)$ and obtained its dependence on $\bar{\rho }$ (Figure 5(a)); we observed a $\left[\left(E_{\bar{\rho }}/E_{s}\right)\propto {\left(\bar{\rho }\right)}^{n},n=2.21\right]$ dependence that matches well with the theoretical analyses on BCC lattice [58]. *d*_33_ and *g*_33_ values measured from the BCC structures not only reveal the well-predicted piezoelectric performance (confirmed by the normalized $\bar{d_{33}}$ that is not sensitive to $\bar{\rho }$ within the linear elastic regime [25, 59]) attributed to consistent printings (Figure 5(b), in Materials and Methods, and Supplementary Section 6) but also validate the remarkably enhanced *g*_33_ (up to *g*_33_ = 460.15 *mV* *m* *N*^−1^) compared to their bulk counterparts (*g*_33_ = 110.71 *mV* *m* *N*^−1^) (Figure 5(c)). With these characterization results, one can affirm the intrinsic superiority of 3D-architected piezoelectric structures as they can simultaneously deliver promoted, tailorable piezoelectric sensing performance as well as mechanical flexibility. To verify that the 3D-printed piezoelectric structures are suitable for self-powered sensing, we printed a set of BCC structures with different f-BTO resins at fixed relative density of $\bar{\rho }=0.17$ and examined their piezoelectric responses under fixed force amplitude *F* = 0.85 *N* (in Materials and Methods and Figure S7). As shown in Figure 5(d), we find that the output voltage *U*_*O*_ can reach up to 44.00 mV, which can be further increased by strategies such as structural design and optimization. All of these results imply that the printed architected piezoelectric structures can meet the practical requirements on self-sustainability put forth by wearable, flexible, and implantable sensing applications [60, 61].

### 2.5. Applications of Printed Piezoelectric Sensors

**Figure 6:**
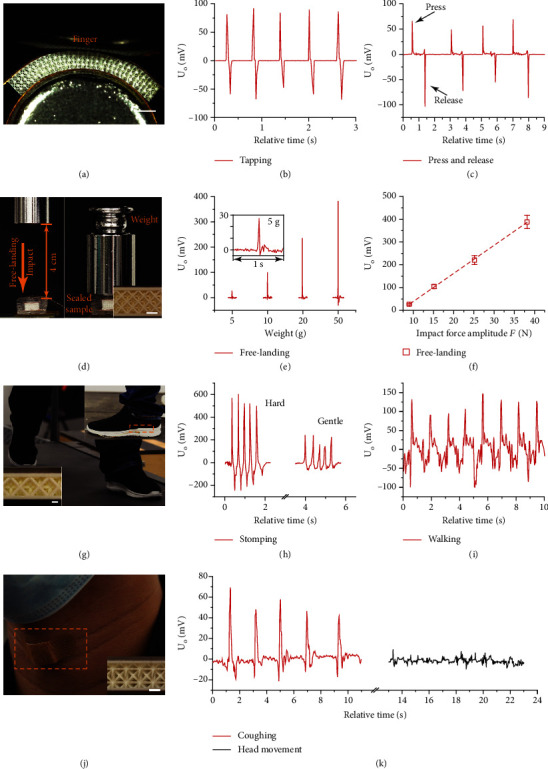
Demonstrations of piezoelectric sensing applications. (a) Optical image of a flexible 30 wt% f-BTO octet-truss lattice structure mounted onto a curved surface (scale bar: 1 mm). (b) and (c) Voltage signals measured during the tapping test and press-and-release test. (d) Schematic of the free-landing impact test. Inset: optical image of the octet-truss structure used in the free-landing test (scale bar: 1 mm). (e and f) Measured output voltage *U*_*O*_. (g) Optical image of the walking test. Inset: optical image of the stomping test; optical image of the octet-truss structure used in the stomping test and walking test (scale bar: 500 *μ*m). (h) Measured output voltage *U*_*O*_ with varying stomping amplitude during the stomping test. (i) Measured output voltage *U*_*O*_ during the walking test. (j) Optical image illustrating the mounted respiratory monitoring device. Inset: optical image of the 3D-printed lattice structure (scale bar: 500 *μ*m). (k) Measured output voltage *U*_*O*_ of the respiratory monitoring sensor under coughing and head movements.

To further demonstrate the sensing capabilities, we printed multiple 3D structures with the 30 wt% f-BTO resin and tested them under a variety of scenarios, schematics of the unit cells were depicted in Figure S14, and associated geometrical parameters were summarized in Table S2. For instance, as shown in Figure 1c2 and Figure 6(a), the 3D-printed architected piezoelectric structures can be easily folded to a great extent and conformally mounted onto curved surfaces due to superior mechanical compliance. The mounted sensor responded swiftly to external stimuli such as tap or press by fingertip and delivered discernible signals (Figures 6(b) and 6(c) and Supplementary Movie 2). We also validated that the piezoelectric sensors could retain their reliable functionalities under scenarios in which integrated package is required. To demonstrate this, a 3D-printed octet-truss lattice structure was sealed with flexible polydimethylsiloxane (PDMS) (Materials and Methods) and mounted atop a load cell, and the sensor was then subjected to free-landing impacts induced by weights, and the real-time output voltage *U*_*O*_ was monitored (Figures 6(d) and 6(e)). The output voltage shows linear dependence versus the impact force amplitude *F* measured by the load cell (Figure 6(f)). It is worth noting that the output voltage can reach up to ~385 mV when the impact force is about 40 N. The results imply that sealings do not deteriorate the effectiveness of the 3D-printed structures as quantifiable, source-free sensors that can deliver instantaneous responses for multipurpose applications once they are calibrated.

To show that the 3D-printed f-BTO structures are suitable for wearable sensing applications, we directly mounted the PDMS-sealed sensor into the shoe insoles (Figures 1(d) and 6(g)) and tested it under foot stompings with varying amplitudes (Figure 6(h) and Supplementary Movie 3) as well as walking test on a treadmill at a fixed pace rate of 0.8 m s^−1^ (Figure 6(i) and Supplementary Movie 4). In particular, the sensor can produce distinguishable signals upon hard and gentle stompings, and during normal walking, the sensor can output a stable and reproducible peak signal of above 100 mV. The stomping test and treadmill test together indicate that the 3D-printed piezoelectric structures can detect and differentiate both static and dynamic human motions; albeit, further device optimization is required to improve the signal-to-noise ratio. We also prototyped a cough monitoring sensor as another proof-of-concept. While coughing, the explosive expansion of the trachea and collective movement of surrounding muscles trigger stimulus that is mimetic to compressive loads, activate the mounted device in *d*_33_ mode to deliver signals recorded for health monitoring and clinical diagnosis (schematic in Figure 1(d)). Here, we chose a highly anisotropic 8-strut node unit with close-to-1 $\bar{d_{33}}$ response and nearly no $\bar{d_{31}}$ or $\bar{d_{32}}$ responses [25] to yield improvement on signal detection as well as suppression on perturbations from daily activities. The packaged sensor was mounted conformally atop the laryngeal of the participant simply via a band-aid (Figure 6(j)). The sensor responded instantaneously with discernible signals, while the participant was coughing and delivered suppressed signals during other daily activities such as head movements (Figure 6(k) and Supplementary Movie 5), and such enhanced sensing performance was attributed to the structural anisotropy owned by the architected piezoelectric structures.

## 3. Discussion

In this work, we present a generalized *μ*CLIP-based 3D printing procedure for rapid productions of architected piezoelectric composite structures. We formulated photo-curable resins comprising commercially available PiezoNPs and yielded printings of sophisticated 3D structures with superior surface finishes at speeds that are at least one order of magnitude faster than other state-of-the-art P*μ*SL-based works. Such superior high-throughput is accessible via the synergetic contributions from underlying advantages of *μ*CLIP as well as chemically functionalized resins with stabilized PiezoNPs dispersions. We conducted systematic studies to verify the tailorable mechanical compliance as well as piezoelectric performance of the 3D-printed piezoelectric structures. We then fabricated multiple piezoelectric sensors using biocompatible f-BTO resins and demonstrated their versatile capabilities under copious scenarios such as impact sensing as well as wearable applications for human motion recognition and respiratory monitoring. We believe the developed procedure serves as a platform that enables the fast fabrication of customized piezoelectric sensing devices as well as the exploration of novel 3D piezoelectric metamaterials capable of delivering anisotropic, directional responses that are both compositionally and structurally tunable. Furthermore, it is evident that the developed procedure is intrinsically suitable for massive productions benefiting from its robustness, high-throughput, multimaterial, and multiscale processibility.

## 4. Materials and Methods

### 4.1. Materials

Chemicals utilized to formulate the resins are all commercially available and used as received. Barium titanate(IV) (nanopowder (cubic), 50 nm (SEM), 99.9% trace metals basis), barium titanate(IV) (nanopowder, cubic crystalline phase, <100 nm particle size, ≥99% trace metals basis), poly(ethylene glycol) diacrylate (PEGDA, average M_n_ 700), 3-(trimethoxysilyl)propyl methacrylate (TMSPMA, 98%), acetic acid (glacial, ACS reagent, ≥99.7%), phenylbis(2,4,6-trimethylbenzoyl)phosphine oxide (Irgacure 819, 97%), and 2-(2H-benzotriazol-2-yl)-6-dodecyl-4-methylphenol (Tinuvin 171) were purchased from Sigma-Aldrich. Ethanol (99.5%, anhydrous, 200 proof, ACROS Organics), 2-propanol (IPA, Certified ACS, Fisher Chemical), and Sylgard 184 silicone elastomer kit were purchased from Fisher Scientific. Lead zirconate titanate (PZT, nanopowder, purity: >99.5%, size: <100 nm) was purchased from Nanografi Nano Technology. Aluminum nitride (AlN, nanopowder, hexagonal, <40 nm) was purchased from Skyspring Nanomaterials. Conductive silver paste was purchased from Ted Pella.

### 4.2. Surface Functionalization of BTO, PZT, and AlN Nanoparticles

~1.5 g BTO nanoparticles were dispersed into 200 mL ethanol, to which 5 mL TMSPMA was added by using a syringe needle. The mixture was sonicated for 1 h, and then, 15 mL of diluted acetic acid solution (10 vol% in water) was added to the mixture prior to functionalization. The mixture was then vigorously stirred at room temperature for 24 h. After that, the functionalized BTO nanoparticles were recollected and cleaned with pure ethanol via centrifugation for at least three cycles. The functionalized BTO nanoparticles were then dried in vacuum at 80°C for overnight and used for preparing the resins. ~1 g PZT or ~ 0.75 g AlN per batch was functionalized via the same procedure as stated above.

### 4.3. Preparation of the Functionalized, Photopolymerizable Resins

f-BTO nanoparticles were dispersed into PEGDA 700 to yield f-BTO weight ratios of 0, 5, 10, 15, 20, 25, and 30 wt%, respectively. Irgacure 819 (photoinitiator) and Tinuvin 171 (photoabsorber) were added to the resins with fixed weight ratios of 2 wt% and 0.2 wt%, respectively. All resins were thoroughly mixed in ultrasonic bath for ~8 h prior to use. 10 and 20 wt% f-PZT resins and 15 wt% f-AlN resins were prepared similarly with fixed 2 wt% Irgacure 819 and 0.2 wt% Tinuvin 171.

### 4.4. Rheological, Morphological, Spectral, Mechanical, and Electrical Characterizations

Rheological and mechanical studies were conducted on a rheometer (Discovery HR-2, TA Instruments) at 25°C. A 40 mm, 2° Peltier cone plate was used for the rheological studies, with a predefined gap thickness of 100 *μ*m for the shear viscosity measurement and 200 *μ*m for time oscillation measurements, respectively, and an oscillation stress of 5 Pa at a frequency of 5 Hz was used for the latter. All mechanical compression tests were conducted with a pair of compression gauges at a constant gauge displacement rate of *μ*m s^−1^. Thermal postcuring on the 3D-printed composites was conducted in a vacuum oven (Across International) at 110°C for 12 h. To extract the bulk Young's modulus *E*_*s*_, round pillars with diameter of 2.5 mm and length of 5 mm were 3D-printed with the f-BTO resins and tested. SEM images and EDS mappings of the 3D-printed structures were obtained by Helios 5 UX SEM/FIB (Thermo Scientific). Raman characterizations were conducted on 3D-printed 1.5 × 1.5 × 5 mm^3^ beam structures via a Raman-AFM-coupled microscope (Alpha300 RA, Witec). Optical images of the 3D-printed structures were taken by a stereomicroscope (SMZ18, Nikon) and a digital camera (Sony A6100). Electrical characterizations on the 3D-printed structures were all conducted via a multimeter (DMM 7510, Keithley) by using proper probes.

### 4.5. Customized *μ*CLIP 3D Printing Setup

Computer-aided design (CAD) software was utilized to design and generate 3D models of the to-be-printed structures. These CAD models were then sliced layer by layer into a series of 2D images by a customized slicing program with predefined layer thickness of 5 *μ*m. A light engine (Pro4500, Wintech Digital) equipped with a 385 nm UV light source and a digital micromirror device (DMD, Texas Instruments) with resolution of 1280 × 800 was used as the optical input to generate the sliced 2D images. A UV lens (UV8040BK2, Universe Optics) was used to project the generated images onto an oxygen-permeable thin film (Teflon AF2400, 40 *μ*m nominal thickness, Biogeneral) embedded underneath a customized resin bath and yields a lateral resolution of 6.9 × 6.9 *μ*m^2^ pixel^−1^ and a maximum lateral printing area of 8.83 × 5.52 mm^2^; all the printings were conducted at a fixed light intensity of 7.6 mW cm^−2^. A CCD camera (MU2003-BI, AmScope) was used to monitor the focusing status of projected images. A *z*-axis-motorized stage (X-LSM200A-KX13A, Zaber Technology Inc.) was used to control the printing platform with varying printing speeds based on predefined printing parameters; a desktop computer was used to control the entire printing procedure.

### 4.6. Corona-Poling and Package of the 3D-Printed Piezoelectric Structures

Corona-poling was carried out on a customized poling setup (Figure S6a), which consisted of a high-voltage power supply (SRS PS375, Stanford Research Systems), a tungsten needle, a copper plate, and a hot plate (Thermo-Fisher Scientific). Temperature of the copper plate surface was set to be 110°C and kept constant during the poling procedures. Distance between the needle tip and top surface of the copper plate (denoted as tip-to-ground distance) was set to be 25 mm, and the poling voltage *V*_poling_ was set to be 20 kV to ensure uniform distribution of electric field *E*_poling_. 3D-printed structures were directly placed on top of the hot plate and beneath the tungsten needle, and the time duration was set to be 1 h. To characterize the piezoelectric responses, both unpoled and corona-poled structures were packaged prior to tests (Figure S6b). Silver paste was applied to top and bottom ends of the printed structures, and copper tapes were leaded and applied as electrodes. The electrodes were then covered with Kapton tapes as surface insulation and protective layers against triboelectricity. Piezoelectric characterization on a set of 30 wt% f-BTO BCC structures by the source-free setup verifies the necessity of corona-poling as well as the effective elimination of triboelectricity, as the poled sample reveals a much higher output voltage (44.00 mV) than the unpoled counterpart (3.74 mV) at a fixed force amplitude *F* = 0.85 *N* (Figure S6c). The PDMS-sealed sensors were prepared by first mixing the Sylgard 184 silicone elastomer base and curing agent with a weight ratio of 10 : 1. The thoroughly mixed precursors were then degassed under vacuum for 2 h. After that, packaged piezoelectric structures were carefully immersed into the precursors and then placed onto an 80°C hot plate for 8 h after secondary vacuum degassing.

### 4.7. Piezoelectric Characterization of the 3D-Printed Structures

The 3D-printed piezoelectric structures were characterized either in a quantitative manner to quantify *d*_33_ and *g*_33_, or in a source-free manner to extract the output voltage *U*_*O*_. For the quantitative piezoelectric charge characterization setup, the packaged structures were placed onto a testing platform, under which a load cell (MLP-10, Transducer Techniques) was connected to measure and record the applied compressive force amplitude *F*. Cyclic compressive loads were exerted onto the piezoelectric structures by a magnetic shaker (LDS V203, Brüel & Kjær) with ramp loading profiles at fixed cyclic frequency *f* of 2 Hz. An amplification circuit consisting of an operational amplifier (OP07CP, Texas Instruments), a feedback resistor *R*_*f*_, and a feedback capacitor *C*_*f*_ was utilized to quantitatively convert the *U*_*O*_ measured by the multimeter during the cyclic tests to charges *q* generated during the cyclic compression tests. The setup was calibrated by using commercially available piezoelectric ceramic discs (APC International) with *d*_33_ values confirmed by a *d*_33_ meter (PolyK) to endow the characterization results (Supplementary Section 3 and Figure S8). In order to extract the bulk *d*_33_ and *g*_33_ of the 3D-printed composites, 8 × 5 × 1 mm^3^ thin film structures were printed and tested. For the source-free characterization setup, the above-mentioned amplification circuit was replaced by a 200 M*Ω* resistor (Schematic diagram in Figure S7).

## Data Availability

All data needed to evaluate the conclusions in the paper are present in the paper and/or the supporting materials.

## Supplementary Materials

Supplementary Materials Supplementary Section 1: determination of optimal printing speed *V*_*s*_. Supplementary Section 2: substrate-assisted printings for resins with high f-BTO loadings. Supplementary Section 3: calibration of the quantitative piezoelectric charge characterization setup. Supplementary Section 4: confirmation of the degree of functionalization of the BTO nanoparticles. Supplementary Section 5: calculations of relative density $\overset{¯}{\rho }$ of 3D-printed BCC structures. Supplementary Section 6: calculations of piezoelectric voltage constant *g*_33_. Figure S1: optical images of the 30 wt% resins prepared with f-BTO and BTO without functionalization (a) right after sonication and (b) 2 d after sonication. Figure S2: (a) oscillation sweep results on the 30 wt% resins prepared with f-BTO and unfunctionalized BTO nanoparticles. (b) Shear viscosity of the 30 wt% resins prepared with f-BTO and BTO without functionalization. (c) Extracted shear viscosity of the f-BTO resins containing different f-BTO loadings at a constant shear rate of 1 s^−1^. Figure S3: schematic and an optical image of the ladder-like model used for determining optimal printing speed *V*_*s*_ (scale bar: 200 *μ*m). Figure S4: schematic of the substrate-assisted printing strategy for 25 wt% and 30 wt% f-BTO resins. Figure S5: measured curing depth *C*_*d*_ versus logarithmic printing speed *V*_*s*_ for the 0 wt% f-BTO resin. Figure S6: (a) schematic of the corona-poling setup. (b) Schematic of the sample package. (c) Output voltage *U*_*O*_ measured from the 30 wt% f-BTO BCC structures ($\overset{¯}{\rho }=0.17$) with and without corona-poling at a fixed force amplitude *F* = 0.85 N. Figure S7: schematic circuit of the source-free characterization setup. Figure S8: (a) schematic of the amplification circuit in the quantitative piezoelectric charge characterization setup. (b) Measured output voltage *U*_*O*_ with two sets of feedback components from a piezoelectric ceramic disc subjected to the same force amplitude F. (c) Calibration result obtained with the feedback components *C*_*f*_ = 300 pF and *R*_*f*_ = 500 M*Ω*. (d) Calibration result obtained with the feedback components *C*_*f*_ = 3000 pF and *R*_*f*_ = 40 M*Ω*. Figure S9: comparisons among *d*_33_ values of the 30 wt% composites printed with f-BTO resins using different amount of BTO nanoparticles during the functionalization. Figure S10: comparisons between *d*_33_ values of the f-BTO composites printed with 50 nm and 100 nm f-BTO. Figure S11: extracted bulk permittivity *ε*_33_ of the f-BTO composites. Figure S12: (a) one representative set of data of the measured charges *q* versus force amplitude *F* for f-PZT bulk composites. (b) Extracted bulk permittivity *ε*_33_ of the f-BTO composites. (c) Extracted piezoelectric charge constant *d*_33_ and piezoelectric voltage constant *g*_33_ of the 3D-printed f-BTO composites. Figure S13: (a) relative density $\overset{¯}{\rho }$ and calculated effective permittivity $\overset{¯}{\varepsilon_{33}}$ of the 3D-printed 30 wt% f-BTO BCC structures with varying average beam diameter $\overset{¯}{D}$. (b) Stress-strain curves of the 30 wt% f-BTO BCC structures with varying relative density $\overset{¯}{\rho }$. Figure S14: schematic of the unit cells used for piezoelectric characterizations and sensing applications, with defined geometrical parameters including unit cell length *L* and beam diameter *D*. Figure S15: comparisons between the measured Young's modulus *E*_*s*_ of the 3D-printed 15 wt% and 25 wt% f-BTO composites with and without thermal postcuring. Table S1: comparisons between the piezoelectric performance of the 3D-printed bulk composites and commonly utilized piezoelectric materials. Table S2: designs and geometric parameters of the lattice structures used for piezoelectric characterizations and sensing applications. Supplementary Movie 1: time lapsed video of the 3D printing of f-BTO composite via *μ*CLIP. Supplementary Movie 2: tapping and press-and-release tests on the 3D-printed structure. Supplementary Movie 3: stomping test on the 3D-printed structure embedded in shoe insoles. Supplementary Movie 4: walking test on the 3D-printed structure embedded in shoe insoles. Supplementary Movie 5: test on the cough monitoring sensor.
