# Supplementary material for: Continuous Three-Dimensional Printing of Architected Piezoelectric Sensors in Minutes
Source: Research (Wash D C). 2022 Jul 11;2022:9790307. doi: 10.34133/2022/9790307 (PMC9318352; doi:10.34133/2022/9790307)
Supplement: Supplementary Materials — Supplementary Section 1: determination of optimal printing speed Vs. Supplementary Section 2: substrate-assisted printings for resins with high f-BTO loadings. Supplementary Section 3: calibration of the quantitative piezoelectric charge characterization setup. Supplementary Section 4: confirmation of the degree of functionalization of the BTO nanoparticles. Supplementary Section 5: calculations of relative density ρ¯ of 3D-printed BCC structures. Supplementary Section 6: calculations of piezoelectric voltage constant g33. Figure S1: optical images of the 30 wt% resins prepared with f-BTO and BTO without functionalization (a) right after sonication and (b) 2 d after sonication. Figure S2: (a) oscillation sweep results on the 30 wt% resins prepared with f-BTO and unfunctionalized BTO nanoparticles. (b) Shear viscosity of the 30 wt% resins prepared with f-BTO and BTO without functionalization. (c) Extracted shear viscosity of the f-BTO resins containing different f-BTO loadings at a constant shear rate of 1 s−1. Figure S3: schematic and an optical image of the ladder-like model used for determining optimal printing speed Vs (scale bar: 200 μm). Figure S4: schematic of the substrate-assisted printing strategy for 25 wt% and 30 wt% f-BTO resins. Figure S5: measured curing depth Cd versus logarithmic printing speed Vs for the 0 wt% f-BTO resin. Figure S6: (a) schematic of the corona-poling setup. (b) Schematic of the sample package. (c) Output voltage UO measured from the 30 wt% f-BTO BCC structures (ρ¯=0.17) with and without corona-poling at a fixed force amplitude F = 0.85 N. Figure S7: schematic circuit of the source-free characterization setup. Figure S8: (a) schematic of the amplification circuit in the quantitative piezoelectric charge characterization setup. (b) Measured output voltage UO with two sets of feedback components from a piezoelectric ceramic disc subjected to the same force amplitude F. (c) Calibration result obtained with the feedback components Cf = [file 9790307.f1.zip › Supplementary Materials_Revised.pdf]

Research
A SCIENCE PARTNER JOURNAL
SPJ
SCIENCE PARTNER JOURNALS

Manuscript

Supplementary Materials

Continuous Three-Dimensional Printing of Architected Piezoelectric Sensors in Minutes

Siying Liu [1,2,3], Wenbo Wang [1,2], Weiheng Xu [1,2], Luyang Liu [1,2], Wenlong Zhang [1,2], Kenan Song [1,2], and ***Xiangfan Chen [1,2,*]***

[1]School of Manufacturing Systems and Networks, Arizona State University, Mesa, AZ 85212, US.

[2]The Polytechnic School, Arizona State University, Mesa, AZ 85212, US.

[3]School for Engineering of Matter, Transport & Energy, Arizona State University, Tempe, AZ 85287, US.

* Email: Xiangfan.Chen@asu.edu

This PDF file includes:

Supplementary Section 1 to 6

Supplementary Figure S1 to S15

Supplementary Table S1 to S2

References

Other Supplementary Materials for this manuscript include the following:

Supplementary Movie 1 to 5

Supplementary Section 1. Determination of optimal printing speed V_s.

A specifically designed ladder-like model consisting of three 100-μm-thick beams (Figure S3) was sliced into a serial of 5-μm-thick images and printed with the f-BTO resins at varying V_s. After printings, measurements based on the optical images were conducted to calculate the measured C_d. Optimal V_s corresponding to the f-BTO resins was defined as the printing speed at which the measured C_d had its value closest to 5 μm and can be directly obtained from Figure 2b as the intersecting points between the dashed line and each curve.

Supplementary Section 2. Substrate-assisted printings for resins with high f-BTO loadings.[1]

A small droplet of 0 wt% f-BTO resin (denoted as substrate resin) was dispensed onto the Teflon film prior to regular printing procedure. The printing platform was then moved downwards to its initial printing position, leading to the flattening of the droplet. The bath was then filled with 25 wt% or 30 wt% f-BTO resin and the regular printing procedure was initiated. The polymerized 0 wt% f-BTO layer (denoted as substrate layer) was mechanically exfoliated after each printing. Schematic was depicted in Figure S4.

Supplementary Section 3. Calibration of the quantitative piezoelectric charge characterization setup.

To ensure the reliability of the extracted d_{33} and g_{33} from the 3D printed composites, appropriate electrical components used in the amplification circuit (Figure S8a) were selected so that 1) the lower limit of characterization frequency f_l is below the cyclic frequency f to ensure consistent and unity gain, 2) relaxation time τ of the feedback loop is sufficiently short to yield transient measurement upon the output voltage U_o.[2,3] 3D printed structures were pre-clamped between the testing platform and the stamp with known clamping forces.[4,5] Upon proper selection of electrical components for the amplification circuit, the generated charges q at a given cyclic force amplitude F can be estimated following

$$U_o \approx -\frac{q}{C_f} \tag{1}$$

where the output voltage U_o was recorded by a multimeter.

To calibrate the established piezoelectric charge characterization setup, two commercially available piezoelectric ceramic discs (APC International) were utilized, whose d_{33} were determined by a commercial d_{33} meter (PolyK) prior to use. Both discs were pre-clamped and subjected to a serial of cyclic loads. The amplification setup was calibrated separately by two sets of feedback components, each with $C_f = 300\,pF$, $R_f = 500\,M\Omega$, and $C_f = 3000\,pF$, $R_f = 40\,M\Omega$, respectively. As shown in Figure S8b, at a given force amplitude $F = 1.46\,N$ the measured average U_0 was ~ 3.37 V and ~ 0.34 V respectively, indicating a ten-fold difference due to the selected feedback capacitors. For each set of the calibration, charges q was plotted versus applied force amplitude F in Figure S8c,d, and the slopes were extracted as the d_{33} values. Extracted d_{33} from both sets showed error less than 5% compared to those obtained by the d_{33} meter. To minimize measurement error by maximizing U_o, the feedback loop with $C_f = 300\,pF$, $R_f = 500\,M\Omega$ was selected for all the subsequent tests on the 3D printed composites.

Supplementary Section 4. Confirmation of the degree of functionalization of the BTO nanoparticles.

To examine whether the BTO nanoparticles in above-mentioned procedure were thoroughly functionalized, another two batches of surface functionalization were conducted with ~ 1.0 g and 2.0 g BTO by keeping the amount of other chemicals (i.e., 5 mL of TMSPMA and 15 mL of 10 vol% acetic acid solution) and processing conditions unchanged. For the ease of presentation here, these f-BTO are denoted by the ratio between BTO and surfactant (TMSPMA). 30 wt% resin was then prepared with these f-BTO, from which f-BTO thin film structures were prepared and characterized. Measured bulk d_{33} values from these 30 wt% thin film structures showed trivial degradation (Figure S9). In detail, for the f-BTO composites processed from BTO / TMSPMA = 1.0 g / 5 mL ,1.5 g / 5 mL and 2.0 g / 5 mL, the d_{33} are $28.03 \pm 1.83\,pC\,N^{-1}$,$27.79 \pm 1.90\,pC\,N^{-1}$ and $26.77 \pm 1.85\,pC\,N^{-1}$, respectively, which are within the margin of error. It indicates that the amount of surfactants used during the functionalization procedure discussed above was far excess than required, hence implies that

the degree of functionalization for 1.5 g per batch was saturated and not able to get further improved.[6]

Supplementary Section 5. Calculations of relative density $\bar{\rho}$ of 3D printed BCC structures.

Optical images of the 3D printed BCC structures were obtained from multiple angles of view to guarantee accurate measurements. Average beam diameter $\bar{D}$ of the 3D printed BCC structures was determined statistically based on the optical images and used as inputs for the reconstruction of CAD models, from which the actual volume V of the BCC structure was obtained. Relative density $\bar{\rho}$ of the BCC lattices was obtained by dividing V by the volume of rectangular envelope of the BCC lattices V_0.[7]

Supplementary Section 6. Calculations of piezoelectric voltage constant g_{33}.

To determine the piezoelectric voltage constant g_{33} of 3D printed f-BTO composites and lattice structures, capacitance C of the f-BTO thin film structures was measured by multimeter, from which bulk permittivity ε_{33} (Figure S11) was extracted following

$$\varepsilon_{33} = \varepsilon_0 \varepsilon_r = \frac{Ct}{lw} \tag{2}$$

where ε_0 is the vacuum permittivity and $\varepsilon_0 = 8.85 \times 10^{-12}\, F\, m^{-1}$, ε_r is the relative permittivity, t, l and w are the measured thickness, length, and width of the thin film structures, respectively. Piezoelectric voltage constant g_{33} was then calculated by

$$g_{33} = \frac{d_{33}}{\varepsilon_{33}} \tag{3}$$

For the case of 3D printed f-BTO BCC structures, effective permittivity $\overline{\varepsilon_{33}}$ (Figure S13a) was calculated following[7]

$$\overline{\varepsilon_{33}} = [\bar{\rho}(\varepsilon_r - 1) + 1]\varepsilon_0 \tag{4}$$

Piezoelectric voltage constant g_{33} of the BCC structures was calculated using modified equation (3) by substituting ε_{33} with $\overline{\varepsilon_{33}}$.

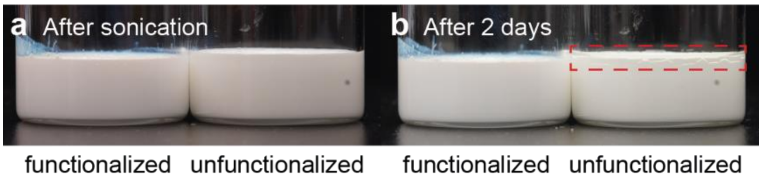

Figure S1. Optical images of the 30 wt% resins prepared with f-BTO and BTO without functionalization (a) right after sonication and (b) 2 d after sonication.

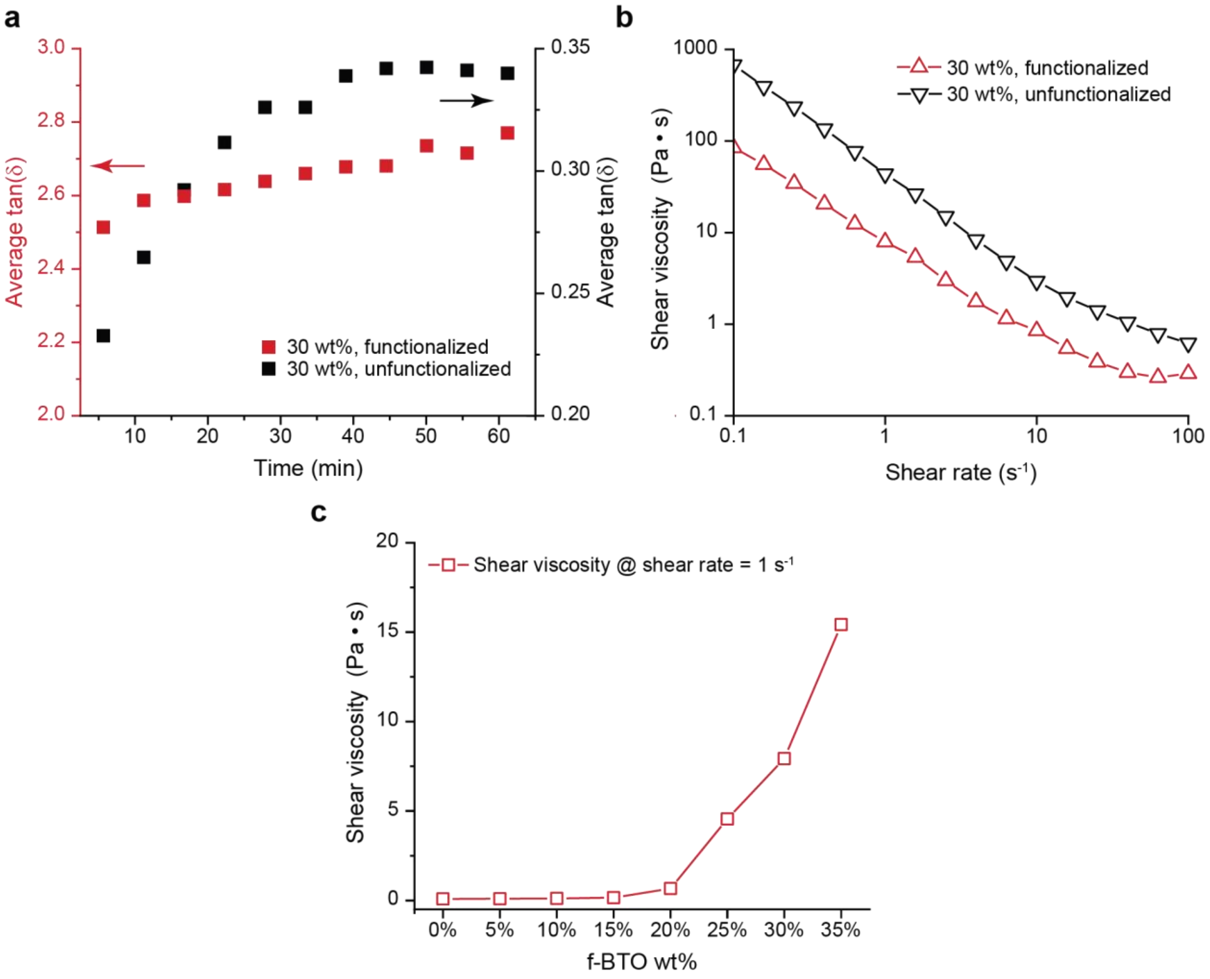

Figure S2. (a) Oscillation sweep results on the 30 wt% resins prepared with f-BTO and unfunctionalized BTO nanoparticles. (b) Shear viscosity of the 30 wt% resins prepared with f-BTO and BTO without functionalization. (c) Extracted shear viscosity of the f-BTO resins containing different f-BTO loadings @ constant shear rate of 1 s^{-1}.

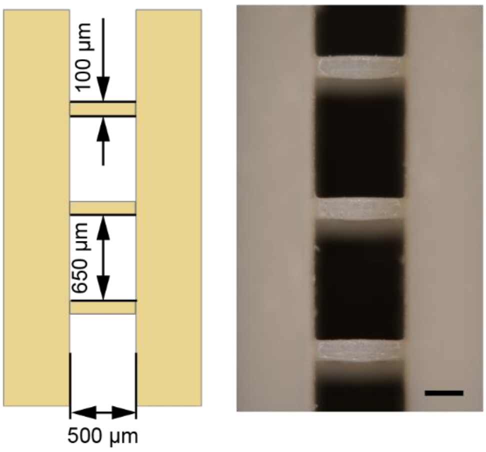

Figure S3. Schematic and an optical image of the ladder-like model used for determining optimal printing speed V_s, scale bar: 200 µm.

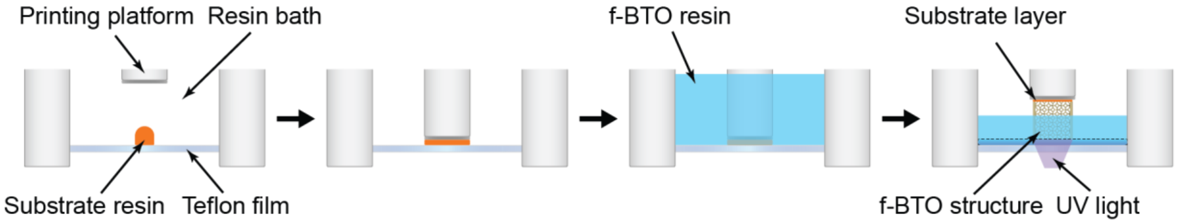

Figure S4. Schematic of the substrate-assisted printing strategy for 25 wt% and 30 wt% f-BTO resins.

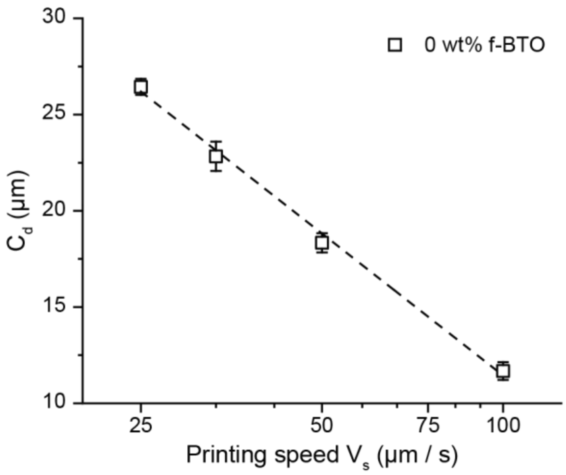

Figure S5. Measured curing depth C_d versus logarithmic printing speed V_s for the 0 wt% f-BTO resin.

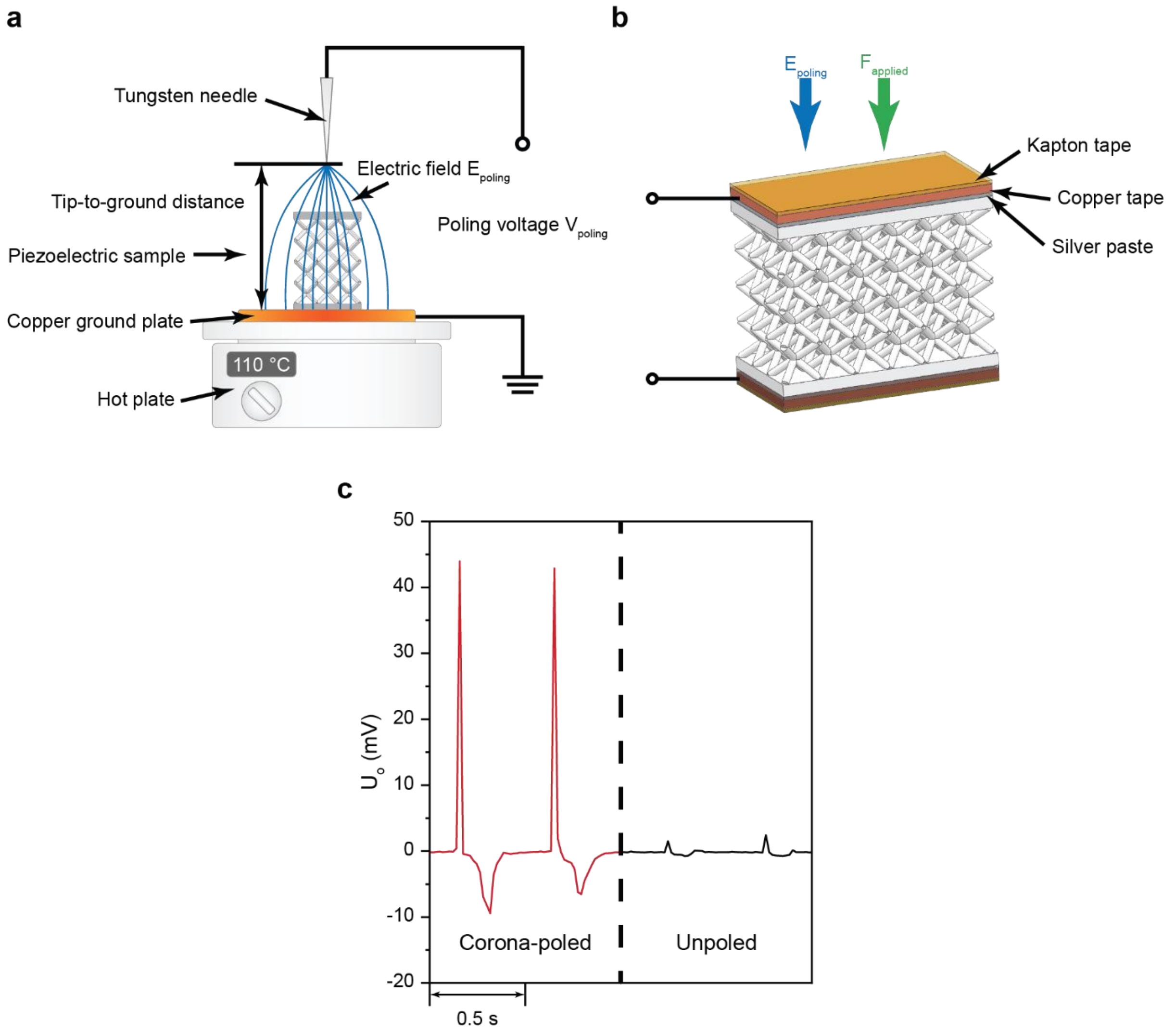

Figure S6. (a) Schematic of the Corona-poling setup. (b) Schematic of the sample package. (c) Output voltage U_O measured from the 30 wt% f-BTO BCC structures ($\bar{\rho} = 0.17$) with and without Corona-poling at a fixed force amplitude $F = 0.85\ N$.

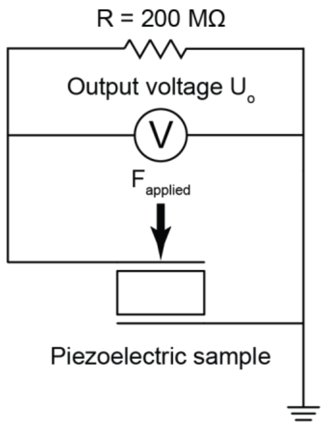

Figure S7. Schematic circuit of the source-free characterization setup.

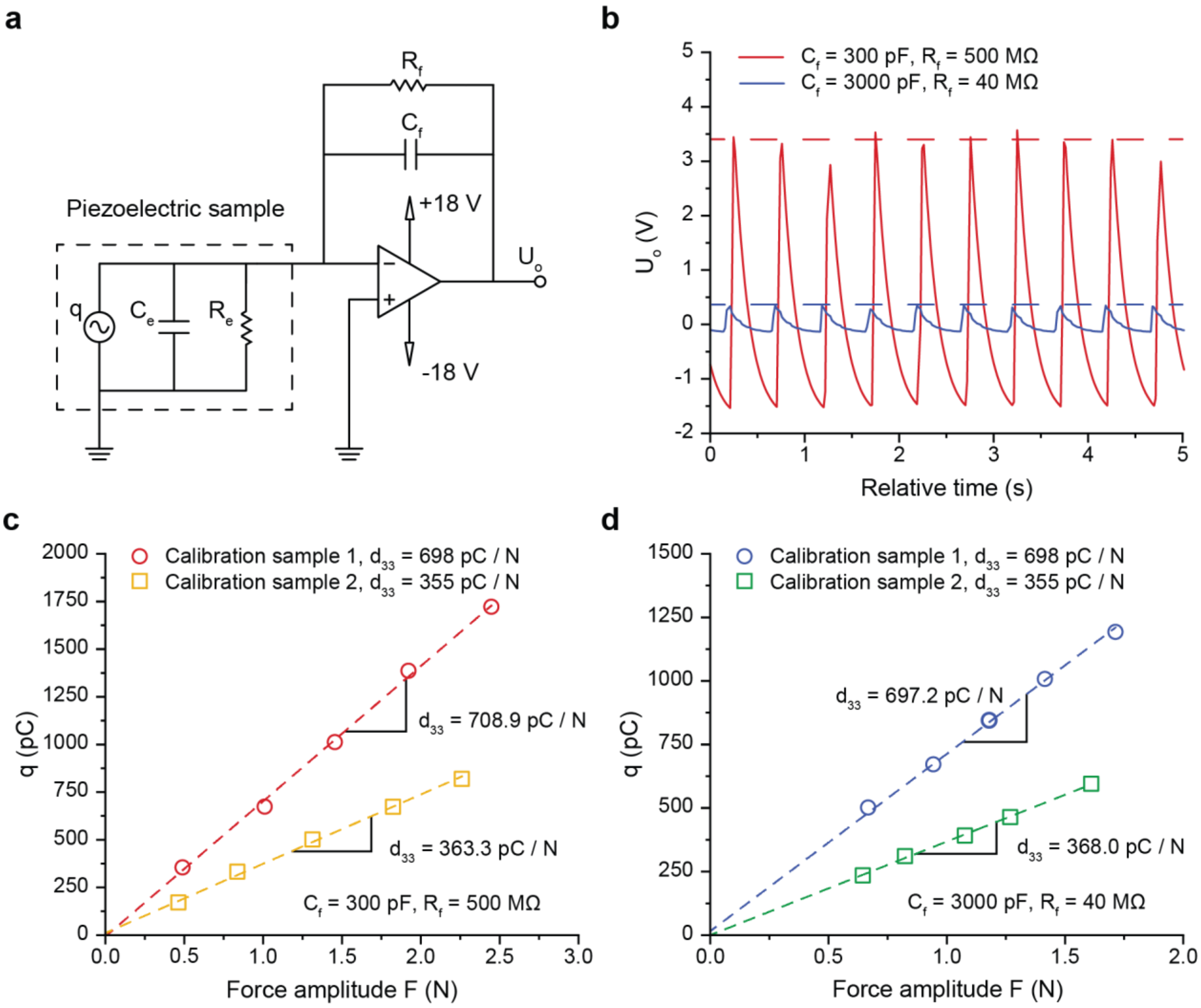

Figure S8. (a) Schematic of the amplification circuit in the quantitative piezoelectric charge characterization setup. (b) Measured output voltage U_O with two sets of feedback components from a piezoelectric ceramic disc subjected to the same force amplitude F. (c) Calibration result obtained with the feedback components $C_f = 300\,pF$, $R_f = 500\,M\Omega$. (d) Calibration result obtained with the feedback components $C_f = 3000\,pF$, $R_f = 40\,M\Omega$.

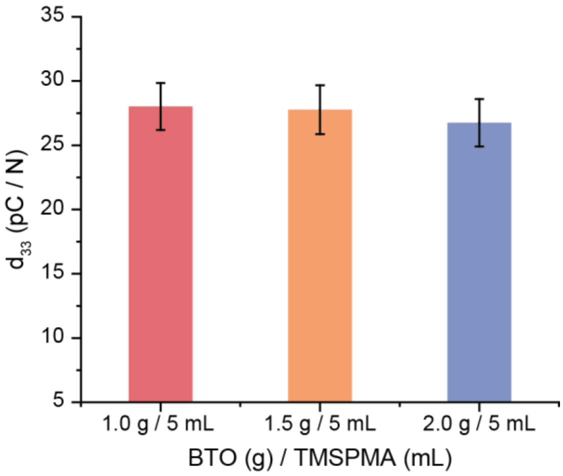

Figure S9. Comparisons among d_{33} values of the 30 wt% composites printed with f-BTO resins using different amount of BTO nanoparticles during the functionalization.

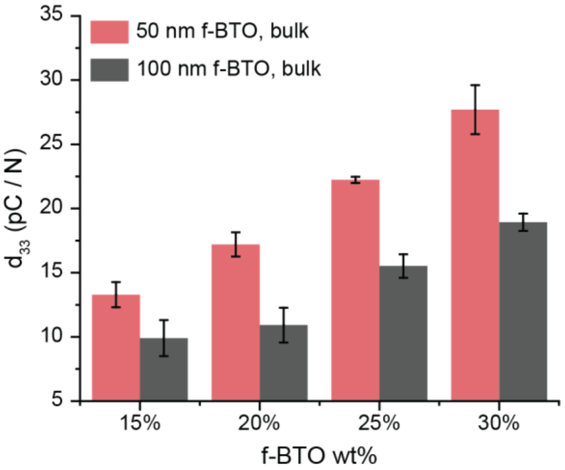

Figure S10. Comparisons between d_{33} values of the f-BTO composites printed with 50 nm and 100 nm f-BTO.

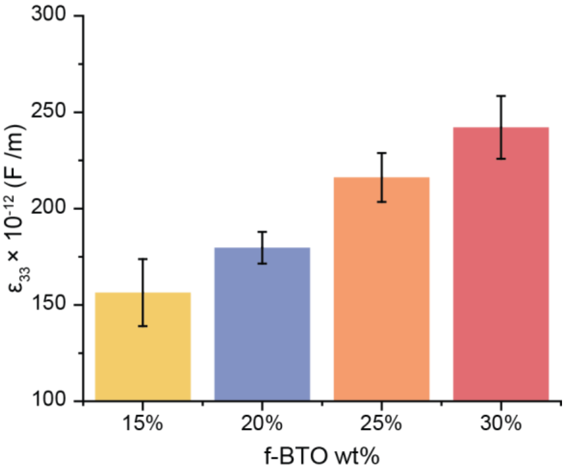

Figure S11. Extracted bulk permittivity ε_{33} of the f-BTO composites.

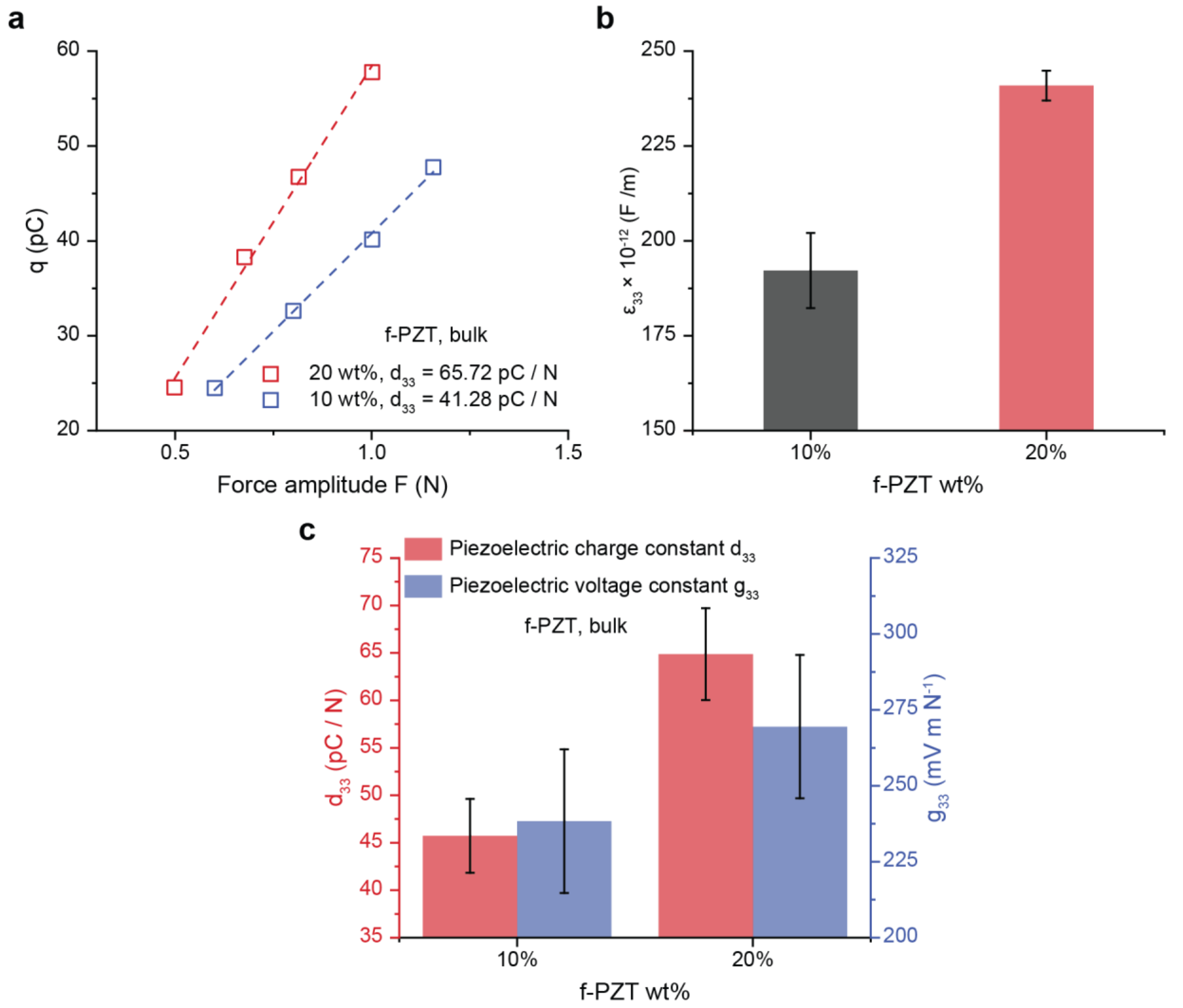

Figure S12. (a) One representative set of data of the measured charges q versus force amplitude F for f-PZT bulk composites. (b) Extracted bulk permittivity ε_{33} of the f-BTO composites. (c) Extracted piezoelectric charge constant d_{33} and piezoelectric voltage constant g_{33} of the 3D printed f-BTO composites.

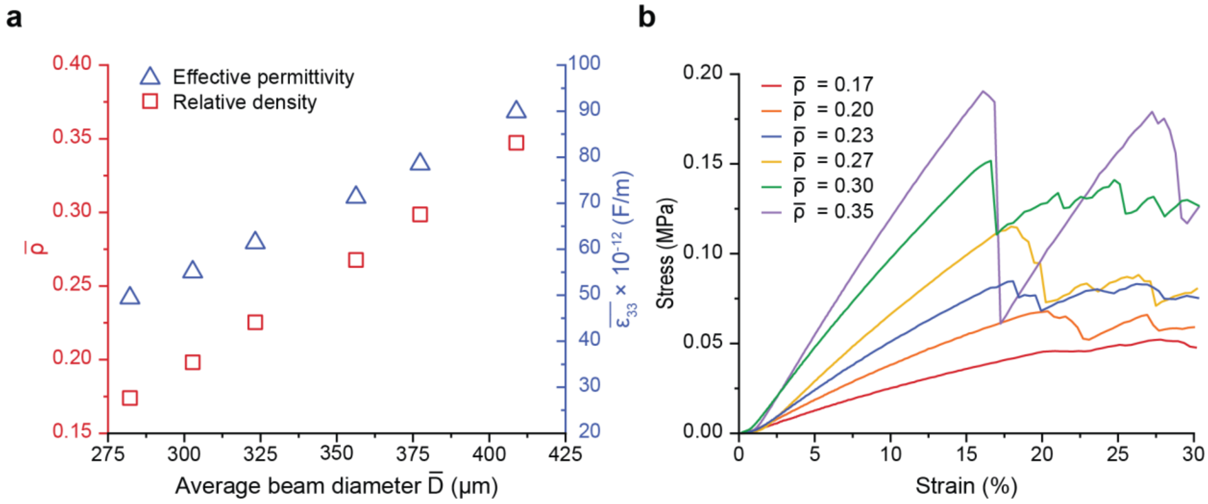

Figure S13. (a) Relative density $\bar{\rho}$ and calculated effective permittivity $\overline{\varepsilon_{33}}$ of the 3D printed 30 wt% f-BTO BCC structures with varying average beam diameter $\bar{D}$. (b) Stress-strain curves of the 30 wt% f-BTO BCC structures with varying relative density $\bar{\rho}$.

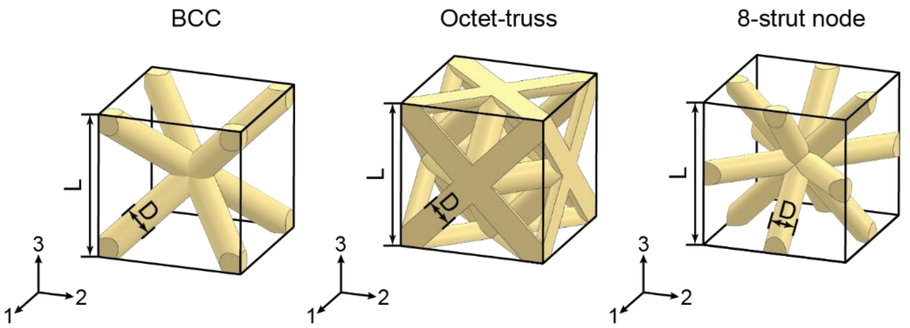

Figure S14. Schematic of the unit cells used for piezoelectric characterizations and sensing applications, with defined geometrical parameters including unit cell length L and beam diameter D.

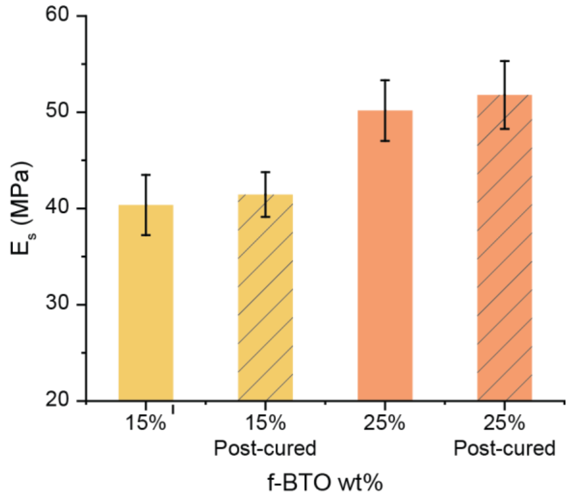

Figure S15. Comparisons between the measured Young's modulus E_s of the 3D printed 15 wt% and 25 wt% f-BTO composites with and without thermal post-curing.

Table S1. Comparisons between the piezoelectric performance of the 3D printed bulk composites and commonly utilized piezoelectric materials.

**Piezoelectric material**	**Type**	**Piezoelectric charge constant d_{33} [pC/N]**	**References**
f-BTO – PEGDA	Composite	12.59 – 28.20	This work
f-PZT – PEGDA	Composite	45.73 – 64.87	This work
PVDF	Pure polymer	-20 – -34	[8]
P(VDF-TrFE)	Pure polymer	-30 – -40	[9]
AlN	Pure ceramic	3 – 6	[10]
BTO	Pure ceramic	190	[11]
PZT – 5H	Pure ceramic	593	[11]

Table S2. Designs and geometric parameters of the lattice structures used for piezoelectric characterizations and sensing applications.

**Scenario of Characterization**	**Unit cell**	**Unit cell length L [μm]**	**Beam diameter D [μm]**
Characterizations on lattice structures	BCC	1500	275, 300, 325, 350, 375, 400
Tapping / Press-and-release	Octet-truss	500	50
Free-landing	Octet-truss	1500	280
Stomping / Walking	Octet-truss	1500	280
Respiratory monitoring	8-strut node[12]	600	100

References

[1] G. Shao, H. O. T. Ware, L. Li, C. Sun, *Adv. Eng. Mater.* **2020**, *22*, 1900911.
[2] G. Gautschi, *Piezoelectric Sensorics*, Springer, Berlin **2002**.
[3] W. Q. Liu, Z. H. Feng, R. B. Liu, J. Zhang, *Rev. Sci. Instrum.* **2007**, *78*, 125107.
[4] Q. Guo, G. Z. Cao, I. Y. Shen, *J. Vib. Acoust. Trans. ASME* **2013**, *135*, 011003.
[5] K. Lefki, G. J. M. Dormans, *J. Appl. Phys.* **1994**, *76*, 1764.
[6] D. Yao, H. Cui, R. Hensleigh, P. Smith, S. Alford, D. Bernero, S. Bush, K. Mann, H. F. Wu, M. Chin-Nieh, G. Youmans, X. Zheng, *Adv. Funct. Mater.* **2019**, *29*, 1903866.
[7] M. L. Lifson, M. W. Kim, J. R. Greer, B. J. Kim, *Nano Lett.* **2017**, *17*, 7737.
[8] H. Kawai, *Jpn. J. Appl. Phys.* **1969**, *8*, 975.
[9] A.J. Lovinger, *Science* **1983**, *220*, 4602.
[10] I. L. Guy, S. Muensit, E. M. Goldys, *Appl. Phys. Lett.* **1999**, *75*, 4133.
[11] R. E. Newnham, *Properties of Materials: Anisotropy, Symmetry, Structure*, Oxford University Press, New York **2005**.
[12] H. Cui, R. Hensleigh, D. Yao, D. Maurya, P. Kumar, M. G. Kang, S. Priya, X. (Rayne) Zheng, *Nat. Mater.* **2019**, *18*, 234.
